# Creutzfeldt-Jakob Disease Presenting with Dementia and Mimic a Stroke During One Year: Case Report and Review of Literatures

**DOI:** 10.31661/gmj.v8i0.1357

**Published:** 2019-01-01

**Authors:** Alireza Vakilian, Mohaddaseh Fekri, Habib Farahmand

**Affiliations:** ^1^Neurology Department, School of Medicine, Rafsanjan University of Medical Sciences, Rafsanjan, Iran; ^2^Non-Communicable Diseases Research Center, Rafsanjan University of Medical Sciences, Rafsanjan, Iran; ^3^Clinical Research Development Center, Ali Ebn Abitaleb Hospital, Rafsanjan University of Medical Sciences, Rafsanjan, Iran; ^4^Radiology Department, School of Medicine, Rafsanjan University of Medical Sciences, Rafsanjan, Iran

**Keywords:** Creutzfeldt-Jakob Disease, Incidence, Dementia

## Abstract

**Background:**

Creutzfeldt-Jakob disease (CJD) is a progressive and fatal prion disease in human and its annual incidence is estimated one per million. Sporadic form of CJD is the most common form of the disease that involved 85% of cases.

**Case Report:**

We presented two cases of CJD with the different clinical presentation; a 58-year-old woman who referred with amnesia, depression and a 59-year-old woman with ataxia as her chief complaint. Based on the findings and roled-out the other differential diagnosis, the CJD was confirmed. Both of them died before 12 months after diagnosis.

**Conclusion:**

Although CJD is a rare disease with different clinical manifestation, it is considered as one the differential diagnosis of progressive dementia.

## Introduction

Creutzfeldt−Jakob disease (CJD) is a neurological disorder that caused by infectious protein substances called prion. CJD is a progressive and fatal disease, which could lead to dementia. Three forms of this disease have become very common recently including the sporadic type involving 85% of CJD cases. The second form is hereditary form caused by mutations (5 to 15% of CJD cases). The third form is the acquired CJD (less than 5%( caused by accidental contamination with surgical instruments, corneal transplantation and dura matter (meningeal) or taking human growth hormone. Contrary to the traditional forms of CJD, one of its variant forms have been reported in younger adults, with the mean age of 28 years old and lasting longer, 14 months on average, in comparison to most of CJD cases (lasting less than one year). The annual incidence of sporadic CJD is 0.5 -1.5 cases per one million that involves an individual over 60 years. It has been seen as sporadic form in old population and a subtype of it in the younger adults [1]. Rapid and progressive dementia is the most common manifestation of sporadic CJD, and it lasts less than one year before death. Due to low incidence, high mortality rate and involvement of different parts of the nervous system, various manifestations have been reported, which led to misdiagnosis. Moreover, the only confirmed diagnosis is reported and published. The first case of CJD in Iran was reported in a hunter in 1999 [2]. Later, other sporadic cases were reported[3-7]. We described two sporadic CJD cases in Rafsanjan city (Kerman Province, with a population of 300,000) within a year while no similar incidences have been previously reported.

## Case Presentation

### Patient 1

**Figure 1:**
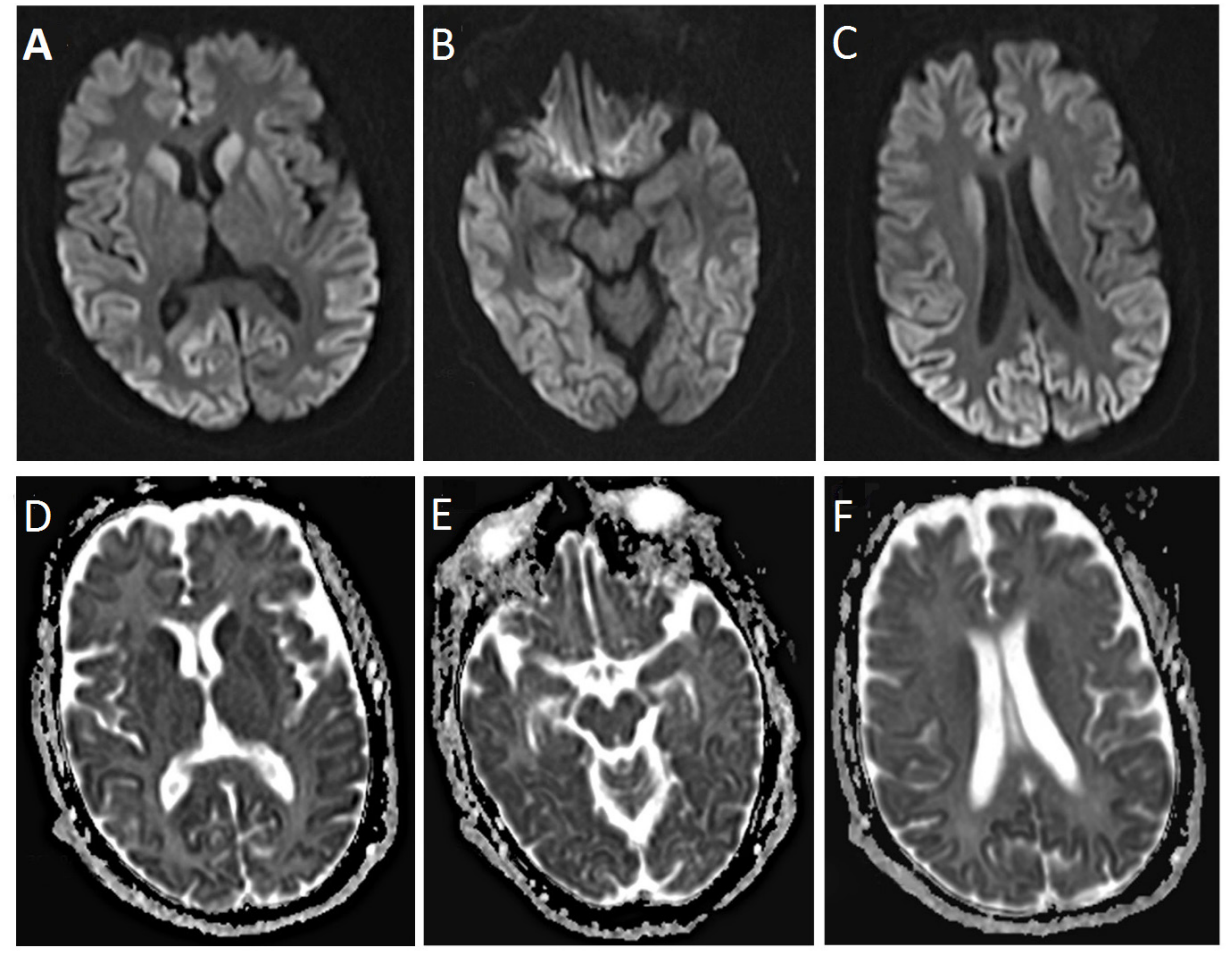
Initial DWI (A-C) and ADC map (D-F) images of patients 1 demonstrate symmetrical restricted diffusion in all of the bilateral cerebral cortices including temporal, frontal, insular, parietal, and occipital lobes and in the bilateral basal ganglia. The brain stem, thalami, and cerebellar hemispheres were spared.

A 58-year-old woman who referred to our clinic (Ali-Ibn-Abitalib Hospital, Rafsanjan, Iran) with dementia and depression symptoms early in 2016. Gradually, her dementia worsened and intensified. Her speech was impaired, and verbal communications decreased. While sleeping, she had twitches and muscle jerks. Her dementia intensified, and her limbs became stiff; she could not maintain her balance while walking. Finally, she lost all her physical strength and was bedridden. Her family members did not agree with that she underwent cerebrospinal fluid (CSF) analysis, while other laboratory data were normal except blood sugar (BS) that was 182 milligrams /deciliter (mg/dl). The brain magnetic resonance imaging (MRI) showed severe restrictions in the diffusion-weighted image (DWI) and apparent diffusion coefficient (ADC) map sequences of all the cerebral hemispheric lobes including frontal, temporal, insular, parietal, and occipital; and symmetric and bilateral restriction in basal ganglia (Figure-1). An increased signal was observed in the T2-weighted sequences of the areas mentioned above. The brain stem, thalami, and cerebellar hemispheres were spared. After intravenous injection of gadolinium, no enhancement was observed in the T1-weighted sequences in the regions mentioned above. Cervical spinal MRI was normal. At the time when mental disorder appeared in her, the periodic waves were observed in Electroencephalography (EEG), which confirmed the disease. The patient died nine months later in the hospital due to aspiration pneumonia and sepsis.

### Patient 2

**Figure 2:**
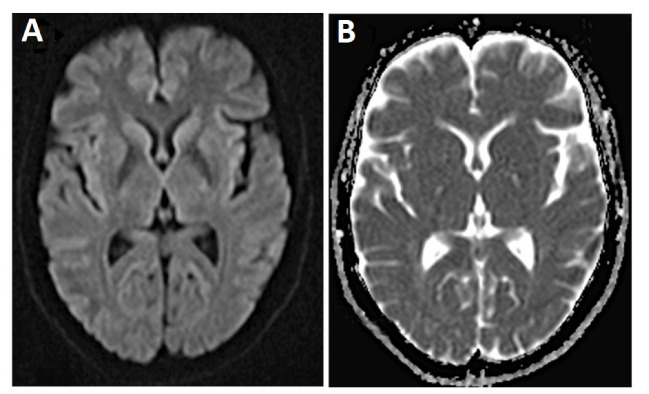
Initial DWI (A) and ADC map (B) images of patient 2 show mild diffusion restriction in the basal ganglia and cortex of bilateral insula.

**Figure 3:**
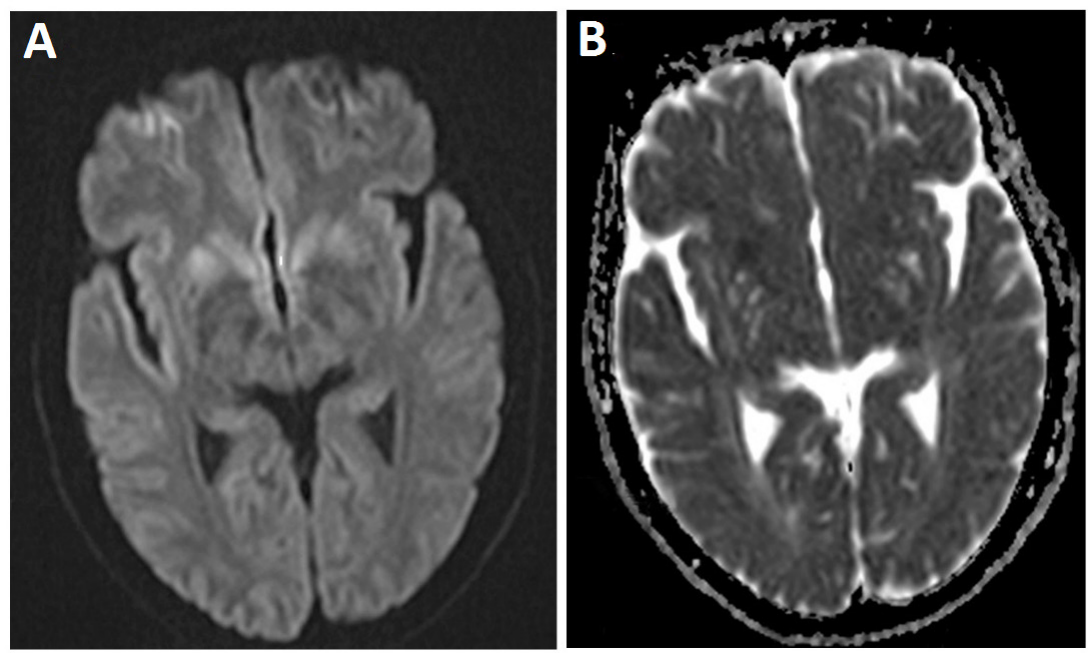
Followed-up MRI of patient 2. DWI (**A**), ADC map (**B**) images demonstrate increased restriction at the head of caudate nuclei and putamen and bilateral insular cortices.

**Figure 4:**
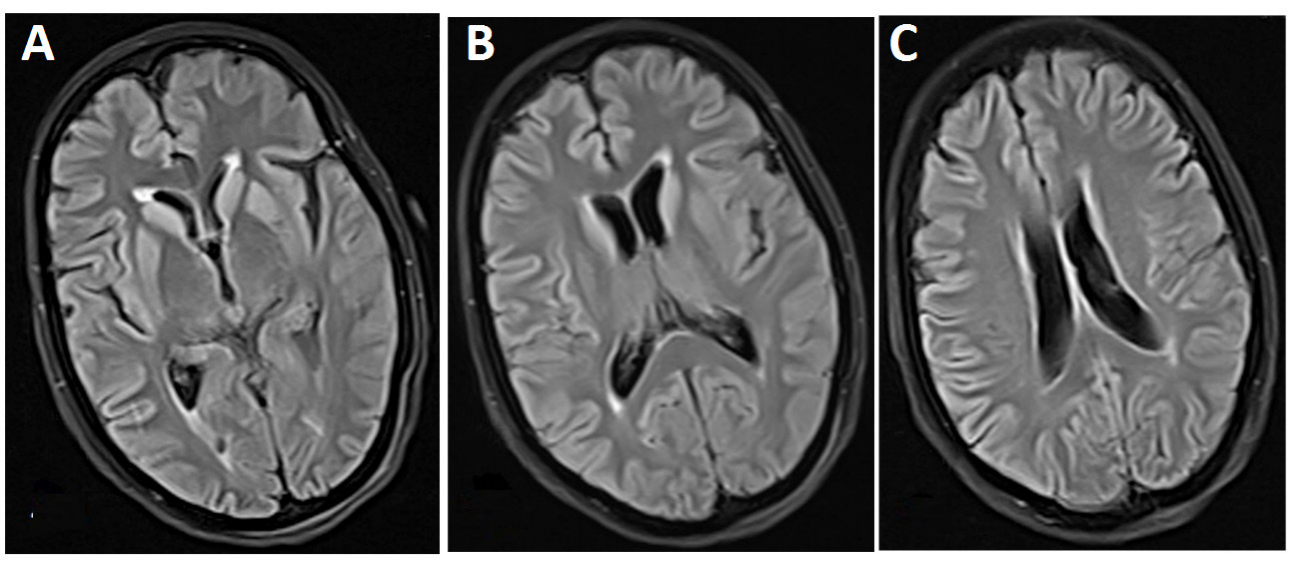
Followed-up FLAIR images of patient 2 reveal symmetric hyperintensity in the basal ganglia (A) and bilateral cerebral cortices (B and C).

**Figure 5:**
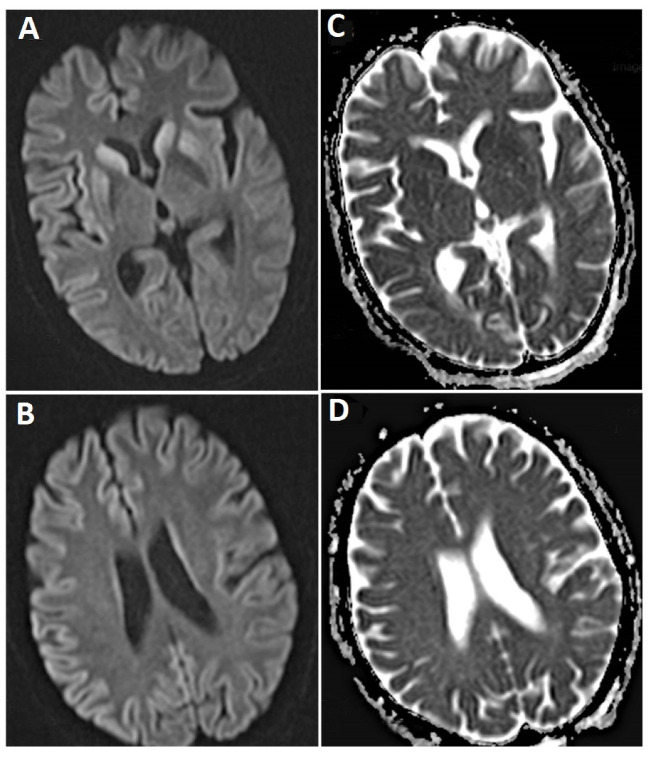
Followed-up DWI (**A** and **B**), ADC map (**C** and **D**) of patient 2 demonstrate increased and persistent restriction in the bilateral basal ganglia and cerebral cortices

A 59-year-old woman who visited with imbalance and ataxia symptoms. At first, neurologists were suspicion of ischemic stroke, but no sign of stroke was observed in MRI. Her laboratory tests were normal except mild leukocytosis (11200 cells/ml) and high BS (142 mg/dl). However, her family members did not agree with CSF examination. Gradually, her walking and memory were impaired; later, she started to have muscle and organ spasms, and reduced communication with her immediate surrounding. Her verbal communication decreased, and she became silent and uncommunicative. She was bedridden, and her swallowing disorder continued leading to aspiration attacks accompanied by trembling, spasms, and organ stiffness once. She was hospitalized on suspicion of central nervous system causes such as acute disseminated encephalomyelitis (ADEM). Computed tomography (CT) scan revealed no any abnormality. In brain MRI, the mild bilateral symmetrical restriction was observed at basal ganglia and insular cortex. (Figure-2). After two weeks, second MRI was conducted that showed bilateral and symmetrical increased signal was in basal ganglia and cortex of frontal, temporal, parietal, as the well as insula lobes in Fluid-Attenuated Inversion Recovery (FLAIR) images and T2-weighted sequences. Increased restrictions are observed in these areas in DWI and ADC map sequences compared to the previous MRI (Figure-3). MRI was done three weeks later showed bilateral and symmetric increased signal in the basal ganglia and cortex of the cerebral hemispheres, which are observed at FLAIR sequences (Figure-4), and increased restriction was seen in the DWI and ADC map sequences (Figure-5). The persistent restriction was the main imaging sign of CJD that was observed in this patient.It should be noted that this patient had various MRI results ranging from mild abnormality to severe one. EEG showed periodic changes in the form of periodic slow waves, which was one of her disease criteria. Finally, she died from aspiration pneumonia and sepsis one year after diagnosis.

## Discussion

CJD is a rare disease; it is a rapidly progressive and neurodegenerative disease that results in death due to neurologic damage [8]. Prion diseases are neurodegenerative disorders that have a long incubation period. Five prion diseases have been discovered in human including Kuru, CJD, variant of CJD, Gerstmann-Straussler-Scheinker syndrome and fatal familial insomnia disease.CJD include 90% of the cases with four forms including sporadic, the acquired, hereditary, and variant forms. The most common symptoms of CJD include rapidly progressive dementia, myoclonus, actinic mutation and symptoms of cerebrovascular disorder [9]. Sporadic CJD is not gendered specific and affects 57-62 years age group[10]. Our patients mentioned above were in this range as well. A 30-100% of CJD incidence is observed in special geographical regions including northern African countries, Italy, Slovakia (the hereditary form). The main manifestations of sporadic CJD include intensified memory disorder and myoclonus. The sporadic CJD consists of several subtypes including MV1, VV1, VV2, MV2, MM2, and MM1. The differential diagnosis of CJD includes autoimmune diseases, infections, malignancies, metabolic and toxic encephalopathy, cerebrovascular diseases, and mental disorder [11]. The clinical criteria for CJD mentioned by the center for preventing and controlling diseases [12] are as follows:

1. Progressive dementia

2. At least two of these symptoms: myoclonus, cerebellar or visual symptoms, pyramidal/extrapyramidal symptoms, and mutism.

3. Abnormal EEG at any stage of the disease or positive 14-3-3 protein in CSF and the disease lasting less than two years leading to death.

4. Abnormal MRI as well as an increased signal in FLAIR and DWI sequences in putamen and caudate nuclei. The best option to diagnose CJD is MRI, which shows abnormal signals of putamen and head of the caudate nuclei. The first symptom of CJD through MRI is increased signal of DWI in the cortex or deep down in the grey matter. CJD is diagnosed by afflicted cornea, larger and progressive putamen and whole atrophy and ventricular dilatation in its delayed phase. Performing MRI frequently cannot be used as a definitive diagnostic tool for CJD; however, studies show that in addition to diagnosis, it helps in monitoring the disease [10]. MRI protocol includes sagittal FLAIR with a 3mm cross section, T2- weighted with 3mm cross section, T1- weighted, and DWI, ADC map. Abnormal MRI results include the increased signal of putamen and head of caudate nuclei, most commonly found in T2- weighted and FLAIR images. One of the best clues and important findings of CJD in MRI is persistent restriction in the DWI, ADC map sequences. Although few cases of CJD have been reported, observed cases involved these results. Less common MRI results include an increased signal at T2- weighted and FLAIR images in globus pallidus, thalamus, hemispheres, cerebellum cortex, and white matter. Laminar damage may be observed in the cerebral cortex and cerebellum. Nevertheless, different results have been reported in different subtypes, and these results can be observed in other diseases. The most sensitive cases are the observed ones in DWI [11]. EEG is a definitive tool for CJD diagnosis. The periodic pattern of sharp two or three-phase wave complexes is seen in 67 to 95% of cases of the sporadic form [10]. Our patients met these CJD criteria; their MRI and EEG results confirmed our findings. Some of the patients have overlapping (similar) symptoms with other diseases like stroke, mental disorders, and Alzheimer disease that leads to accurate diagnosis take more time. Some of the patients had been misdiagnosed, and they underwent the maltreatment; thus, the disease developed and clinical suspicion changed the direction towards new diagnostic procedures, and they were diagnosed with CJD eventually. Patients presented in this study were sporadic CJD. One important point in CJD diagnosis is an overlap of its symptoms with other diseases, and it is not possible to diagnose CJD by its symptoms before excluding all other diseases that have a similar presentation. On the other hand, CJD is less common in comparison to another differential diagnosis of it. This is one of the challenges for detecting a patient suffering from CJD. In Iran, CJD cases have been reported in Mashhad, Tehran, Tabriz, Isfahan and Ahvaz cities; one of these cases was hereditary CJD [2-7]. The first case of CJD in Iran was reported in a 53-year-old hunter in 1994 by Joneidi et al.; his symptoms included reduced memory stability, lack of orientation, monotonous speech, and stiffness of the organs. These symptoms gradually changed into periodic laughter and cries out of restlessness, urinary incontinence, reduced verbal communication, myoclonus, and seizure [2]. Nikkhah et al. (1996) reported a 70-year-old man with a speech disorder, general weakness, myoclonus, abnormal behaviors and memory loss [3]. Nikfar et al. (2004) presented 71-year old women with visual symptoms, transient confusion, disorientation, dystonia in the right side [4]. Ghorbani et al. (2007) reported a 56-year-old woman in with confusion, delusion, rigidity, dysphagia, and myoclonus [5]. Sarraf et al. (2014) presented 50-year-old women with cognition problems, mutism, imbalance and tinnitus presentation [6]. Shaygannejad et al. (2007) reported a 61-year-old man with impaired cognition function, hallucination, rigidity, myoclonus jerk and ataxia [7]. The symptoms of our patients started and developed gradually. We, initially, suspected stroke and other causes of dementia and imbalance. MRI and EEG findings after three months helped us to diagnose the disease accurately, and slowly their symptoms met CJD criteria.

## Conclusion

Numerous diagnostic procedures have been introduced for CJD diagnoses that require the consent of the patients’ family members and the necessary equipment in the disease-afflicted area. Due to the low incidence of CJD, it should be placed at the bottom of the diagnosis list and chosen after rejecting other diseases. The best cues to detect a CJD patient is the progressive and incapacitating nature of the disease that leads to death eventually.

## Acknowledgment

The authors would like to thank the Clinical Research Development Center of Ali-Ibn Abi Talib Hospital for its supports.

## Conflict of Interest

All authors reviewed the article, and they have agreed with the contents of the paper. There are no conflict of interest regarding this paper.
